# Fucosylated Human Milk Oligosaccharides during the First 12 Postnatal Weeks Are Associated with Better Executive Functions in Toddlers

**DOI:** 10.3390/nu15061463

**Published:** 2023-03-17

**Authors:** Yvonne Willemsen, Roseriet Beijers, Fangjie Gu, Alejandro Arias Vasquez, Henk Arie Schols, Carolina de Weerth

**Affiliations:** 1 Department of Cognitive Neuroscience, Donders Institute for Brain, Cognition and Behaviour, Radboud University Medical Center, 6525 EN Nijmegen, The Netherlands; 2 Behavioural Science Institute, Radboud University, 6525 GD Nijmegen, The Netherlands; 3 Laboratory of Food Chemistry, Wageningen University & Research, 6708 WG Wageningen, The Netherlands; 4 Donders Center for Medical Neuroscience, Department of Psychiatry and Human Genetics, Radboud University Medical Center, 6525 GA Nijmegen, The Netherlands

**Keywords:** human milk oligosaccharides, executive functions, longitudinal, preschool

## Abstract

Human milk oligosaccharides (HMOs) are one of the most abundant solid components in a mother’s milk. Animal studies have confirmed a link between early life exposure to HMOs and better cognitive outcomes in the offspring. Human studies on HMOs and associations with later child cognition are scarce. In this preregistered longitudinal study, we investigated whether human milk 2′-fucosyllactose, 3′-sialyllactose, 6′-sialyllactose, grouped fucosylated HMOs, and grouped sialylated HMOs, assessed during the first twelve postnatal weeks, are associated with better child executive functions at age three years. At infant age two, six, and twelve weeks, a sample of human milk was collected by mothers who were exclusively (*n* = 45) or partially breastfeeding (*n* = 18). HMO composition was analysed by use of porous graphitized carbon-ultra high-performance liquid chromatography–mass spectrometry. Executive functions were assessed at age three years with two executive function questionnaires independently filled in by mothers and their partners, and four behavioural tasks. Multiple regression analyses were performed in R. Results indicated that concentrations of 2′-fucosyllactose and grouped fucosylated HMOs were associated with better executive functions, while concentrations of grouped sialylated HMOs were associated with worse executive functions at age three years. Future studies on HMOs that sample frequently during the first months of life and experimental HMO administration studies in exclusively formula-fed infants can further reveal associations with child cognitive development and uncover potential causality and sensitive periods.

## 1. Introduction

Human milk is considered the best nutrition for an infant because of its beneficial effects on child development and on maternal and child health [1]. For example, human milk is shown to protect against infections during infancy and metabolic diseases in later life [2]. Moreover, breastfeeding parameters, such as the initiation and duration of breastfeeding, have been related to improved child neurodevelopment and cognition [2,3]. Increasing evidence also shows that not only breastfeeding parameters, but also specific constituents of human milk, are related to child behavioural and cognitive outcomes (for a review see de Weerth et al. [1]). This concept of human milk determining the trajectory of development with long-term consequences for the phenotype is also known as Lactocrine Programming [4,5,6]. Human milk oligosaccharides (HMOs) are the most abundant solid component in human milk after lipids and lactose [7]. With respect to Lactocrine Programming, HMOs are of interest because of their potential beneficial effects on child neurodevelopment [7,8]. However, to date, the few studies that investigated HMOs’ associations with child cognition focused on general cognition and language, and reached the age of 24 months [9,10,11,12]. The current study extends these first findings by investigating, in a healthy community sample, the longitudinal associations between HMOs in the first weeks of life and executive functions, which represent higher cognitive abilities, at a child age of three years.

HMOs are complex carbohydrates made up of various combinations of five monosaccharides (i.e., galactose, glucose, *N*-acetylglucosamine, fucose, and sialic acid). Based on combinations of these monosaccharides, HMOs can be divided into three groups: neutral core HMOs (containing only glucose, galactose, and *N*-acetylglucosamine residues), fucosylated HMOs (containing a lactose or neutral core backbone, with one or more fucose units), and sialylated HMOs (containing a lactose or neutral core backbone with one or more sialic acid units) [13]. The HMO structure determines the HMO function and influence on the body [14]. Women can secrete about 200 different structures of HMOs, though 10 individual HMOs make up over 70% of the total HMO concentration [15]. While HMO composition remains mainly constant during the day and week, HMO composition does change throughout lactation and varies greatly between women [15,16,17]. One of the most important factors explaining variance in milk HMO composition is secretor status. Secretor status is controlled by the FUT2 gene and refers to the presence or absence of water-soluble ABO blood group antigens in a person’s bodily fluids, including breast milk. People who secrete these antigens in their bodily fluids are referred to as secretors, while people who do not are termed non-secretors. Maternal secretor status has been shown to affect levels of HMOs with fucose-containing structures [18]. Mothers who are secretor-negative usually produce none, or very low levels of 2′-fucosyllactose (2′FL), as opposed to secretor-positive mothers [18].

Because the most important function of HMOs is to provide nutrients to specific gut bacteria, the microbiota-gut-brain axis is a likely pathway through which HMOs can ultimately exert effects on the brain and behaviour. Rodent and human studies have shown that the microorganisms in our gut, or gut microbiota, are able to communicate with the brain via the microbiota-gut-brain axis, which is the bi-directional communication route between the gut microbiota and the brain [19,20]. Mainly bifidobacteria benefit from HMOs; for example, *Bifidobacterium longum* subspecies *infantis* uses HMOs as metabolic substrates [21,22,23]. The exact mechanism of how bifidobacteria can subsequently affect brain development is still unclear. However, bifidobacteria are able to produce short-chain fatty acids, which are able to cross the blood–brain barrier and exert positive effects on the brain [24]. This proposed mechanism may explain associations between HMOs and cognitive development.

HMOs have been causally related to long-term cognition in animal studies (for a review on HMO administration in animal studies, see Docq et al. [25]). For example, 2′FL administered at early ages (in combination with other components or HMOs) until the end of the experiment (postnatal day 33) contributed to improved memory performance and faster learning speed in adult pigs, compared to control pigs [26,27]. Furthermore, sialylated HMOs, mainly 3′-sialyllactose (3′SL) and 6′-sialyllactose (6′SL), administered at an early age until the end of the experiment (postnatal day 19 and 35) contributed to better performance of rats and piglets in memory and learning tasks in adolescence and older adulthood, compared to control animals [28,29]. In addition, Oliveros et al. [30,31] administered 2′FL and 6′SL in rats in early life only and found an association with better performance on learning tasks in adulthood. These positive effects of early-life HMO administration on memory and learning in adulthood indicate that HMO consumption in early life can exert lifelong effects on the cognition of mammals.

Mechanisms have been explored for several HMOs. The HMO 2′FL is known for its specific stimulation of the growth of bifidobacteria in the gut [32]. Additionally, in rat brains, 2′FL induced long-term potentiation (LTP) which is involved in learning and memory [33,34,35]. Next to that, deprivation of 6′SL affected cognitive functions, as seen by reduced expression of important genes in the prefrontal cortex, a brain region that mediates executive functions and memory [36]. Lastly, sialic acid has been suggested to play a key role in neurodevelopment during the early postnatal stages, as it provides the building blocks for brain gangliosides [37], which have been related to neurophysiological outcomes, such as memory formation [8,37,38].

To our knowledge, no studies on the effects of HMO supplementation on cognition have been performed in humans. However, observational human studies are emerging that investigate longitudinal relations between HMOs, cognition, and related constructs. One study found that 2′FL concentrations in milk samples collected at 1 month postpartum were associated with better cognitive development at 24 months of age, as measured with the Bayley Scales of Infant and Toddler Development [10]. Another longitudinal human study found a positive association between 6′SL and better cognitive development scores at 18 months of age, also measured with the Bayley Scales [9]. In addition, Cho et al. found positive links between 3′SL concentration and a composite of cognition at multiple ages (language in particular) in human infants [11]. Lastly, a large study in Malawi participants found a positive link between grouped fucosylated and grouped sialylated HMO concentrations in mothers’ milk collected at 6 months postpartum, and language at child age 18 months [12]. To summarise, most human studies assessed HMO concentrations only at one time point or at older ages (i.e., 6 months). Because HMO composition changes over lactation and early life is known to be a sensitive period for future child development, multiple samples in early life are required to obtain a more reliable picture of an infant’s exposure to HMOs during the first months of life and its association with later child cognitive development.

This prospective longitudinal study investigated, in a healthy Dutch community sample, the associations between HMOs measured at infant age 2, 6, and 12 weeks, and executive functions at child age 3. As inhibitory control is an important aspect of executive functions and is essential for child mental health development [39,40], we also included behavioural measures of inhibitory control. The study was preregistered on the Open Science Framework (https://doi.org/10.17605/OSF.IO/H4ZTW, registered on 3 May 2022). Based on the literature mentioned above, we selected specific HMOs (2′FL, 6′SL, and 3′SL) and composed two HMO groups (fucosylated- and sialylated HMOs) for our study. We hypothesized that 2′FL, 6′SL, 3′SL, grouped fucosylated HMOs, and grouped sialylated HMOs would be positively associated with better executive functions and inhibitory control. With the goal of expanding our knowledge on HMOs, we exploratorily investigated associations between child cognition and 21 other HMOs: 3′-fucosyllactose (3-FL), difucosyllactose (DFL), di-/tri-fucosyllacto-N-hexaose (DF-/TF-LNH), four different isomers of fucosyllacto-N-hexaose (F-LNH), isofucosyl-Lacto-N-hexaose I (IF-LNH-I), lacto-N-difucohexaose I (LNDFH-I), lacto-N-difucosylhexaose II (LNDFH-II), lacto-N-fucopentaose I (LNFP-I), lacto-N-fucopentaose II (LNFP-II), lacto-N-fucopentaose III (LNFP-III), lacto-N-fucopentaose V (LNFP-V), lacto-N-tetraose (LNT) and lacto-N-neotetraose (LNnT) combined, lacto-N-hexaose (LNH), lacto-N-neohexaose (LNnH), para-lacto-N-hexaose (pLNH), sialyllacto-N-tetraose-a (LSTa), sialyllacto-N-tetraose-b (LSTb), sialyllacto-N-tetraose C (LST c), and three more HMO groups: non-fucosylated and non-sialylated HMOs, mono-fucosylated HMOs, and di- and tri-fucosylated HMOs.

## 2. Materials and Methods

### 2.1. Participants

**Figure 1:**
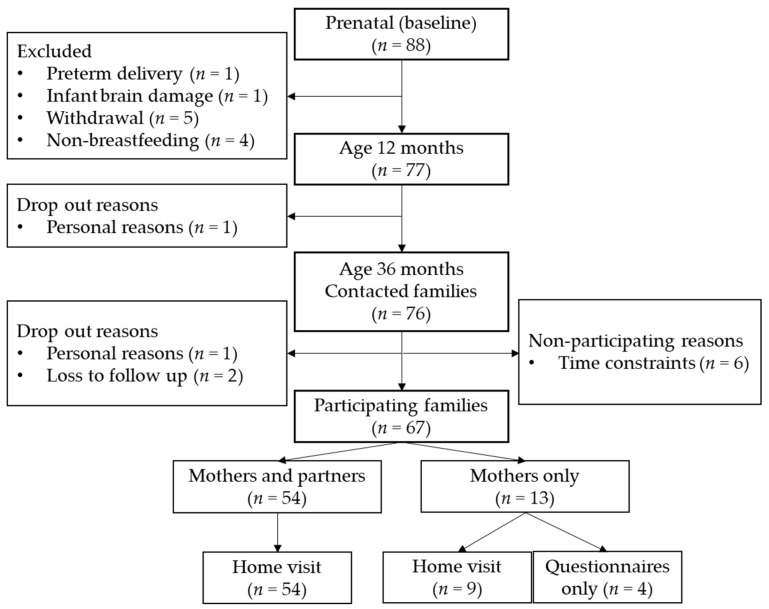
Flowchart of participant follow-up. Drop-outs are shown on the left side of the flowchart. Participants who skipped the assessment round are shown on the right side of the flowchart.

This study is part of the longitudinal *BINGO* study investigating early prenatal and postpartum predictors of child development [41,42]. Participants were parents and their children, recruited in the Netherlands during pregnancy in 2014/2015 (*n* = 88). Postnatal exclusion criteria were pregnancy complications, birth weight <2500 g, gestational age at birth <37 weeks, 5 min Apgar score <7, and congenital malformations [41]. After birth, eleven participating families were excluded, either because inclusion criteria were not met, or withdrawal due to personal circumstances. After one drop-out during the first postnatal year, 76 families were contacted at the 36-month measurement round (2017/2018) and 67 agreed to participate (see participant flowchart in Figure 1). Of these 67 participants, 4 mothers did not breastfeed during the first postnatal weeks and thus did not provide milk samples, resulting in a total number of 63 participants for the current study. All the analyses were first performed on data from exclusively breastfed children during the first 12 postnatal weeks (*n* = 45, including two infants who received one formula feeding a week) to avoid potential effects of formula feeding on behaviour. Subsequently, the analyses were repeated with the whole group of infants (*n* = 63), correcting for percentage breastfeeding (see HMOs section).

The *BINGO* study was independently reviewed by the Ethics Committee of Social Sciences (ECSW) of Radboud University, and no formal objection to this research was made [ECSW2014-1003-189 and amendment: ECSW-2018-034]. The current study was preregistered on the Open Science Framework: https://doi.org/10.17605/OSF.IO/H4ZTW, registered on 3 May 2022.

### 2.2. Procedure

Mothers collected milk samples at two, six, and twelve weeks after delivery. Mothers collected these samples (approximately 20 mL) into sterile collection cups by hand expression in the morning, before feeding the infant. Mothers were asked to wash their hands before collection. In case breast pads or cream had been used, mothers were asked to also wash their breasts before collection. The three mothers who collected milk via a pump were asked to first boil the parts that come into contact with the milk. Samples were stored in the participant’s freezer before they were collected with a portable freezer when the infant was around 12–14 weeks. The samples were subsequently stored at −80 °C at the Radboud University, and afterwards delivered to the lab of Food Chemistry of Wageningen University for HMO content analysis.

At three years of age, mothers and their partners independently filled in online questionnaires about their child’s executive functioning. In addition, home visits took place, where child inhibitory control was assessed by a trained examiner through six inhibitory control tasks. For more information on the procedure and content of the visit, see Willemsen et al. [43].

### 2.3. HMOs

The HMOs were extracted, purified by solid phase extraction, and quantified by using porous graphitized carbon-ultra high-performance liquid chromatography–mass spectrometry (PGC-UPLC-MS) and high-performance anion exchange chromatography with pulsed amperometric detection (HPAEC-PAD) [44]. The following 24 different HMO structures were determined: 2′FL, 3-FL, 6′SL, 3′SL, DF-TF-LNH, DFL, four different isomers of F-LNH, IF-LNH-I, LNDFH-I, LNDFH-II, LNFP-I, LNFP-II, LNFP-III, LNFP-V, LNH, LNnH, pLNH, LNT and LNnT combined, LST a, LST b, and LST c (see also Borewicz et al. [17] and Gu et al. [45] for other analyses with these data).

The group of fucosylated HMOs consisted of 3-FL, 2′FL, LNFP-I, LNFP-II, LNFP-III, LNFP-V, four isomers of F-LNH, IF-LNH-I, LNDFH-I, LNDFH-II, DFL, and DF-TF-LNH. The group of sialylated HMOs consisted of 6′SL, 3′SL, LST a, LST b, and LST c. The group of non-fucosylated and non-sialylated HMOs consisted of LNT and LNnT combined, LNH, LNnH, and pLNH. The group of mono-fucosylated HMOs consisted of 3-FL, 2′FL, LNFP-III, LNFP-II, LNFP-I, LNFP-V, four different isomers of F-LNH, and IF-LNH-I. The Di- and Tri-fucosylated HMOs consist of LNDFH-I, LNDFH-II, DFL, and DF-TF-LNH. The identification of different LNH isomers was not possible as the pure substances for identifying LNH isomers were not commercially available during the time of wet analyses. However, identification of LNH isomers was achieved based on mass-to-charge ratios of peaks and was then compared to retention times in the literature. This allowed for relative comparisons between the HMOs.

Due to naturally occurring differences in milk dilutions, HMO concentrations were corrected for sample-to-sample variability by normalizing readout values for each time point separately, using the Probabilistic Quotient Normalization (PQN) method in R, as performed by Borewicz et al. [17]. Furthermore, corrections for estimated daily milk intake were based on previous literature (480 g, 580 g, and 630 g at weeks two, six, and twelve, respectively) [46]. The resulting variables were used as such for the exclusive breastfeeding group (*n* = 45). For the total group (*n* = 63), in which also mothers were included who were partially breastfeeding, these corrected values were further adjusted for the proportion of human milk feedings. E.g., if the infant at 12 weeks received 30% formula and 70% human milk, the corrected HMO concentrations at that time point were further adjusted by multiplying by 0.7.

### 2.4. Executive Functions

Two questionnaires were used to measure child executive functioning. The Behavior Rating Inventory of Executive Function-Preschool Version (BRIEF-P) is a commonly used executive function questionnaire that measures general child executive functions and does not differentiate between different situations. The Ratings of Everyday Executive Functioning (REEF) is less commonly used and rates child executive functions in different situations (e.g., executive functions around friends, during grocery shopping, or in the community). Because of their different assessment methods, and because previous literature showed different outcomes between the two questionnaires [43], we included both in our research.

The BRIEF-P [47] is a 63-item questionnaire that assesses preschool-aged executive functioning using a 3-point scale (option answers: ‘Never’, ‘Sometimes’, ‘Often’). Example items are: “Overreacts to small problems” and ”Is easily overwhelmed or overstimulated by typical daily activities.” Higher scores indicate worse executive functioning. To align with our other executive functioning and inhibition measures, the outcome of the BRIEF-P was reverse-coded. Consequently, higher scores on the BRIEF-P indicated better executive functioning. The Cronbach’s alpha was 0.94 for mothers and 0.96 for partners.

The REEF [48] is a 77-item questionnaire that assesses preschool-age executive functions using a 4-point scale (option answers: ‘Is not able’, ‘Never or almost never’, ‘Sometimes’, ‘Always or almost always’). This questionnaire assesses the child’s behaviour in eight different scenarios, namely: how the child plays games, how the child plays games with others, how the child interacts with others, around the house, in the community, out shopping, story time, and general skills and behaviours. Example items are: “Plays “Hide and Go Seek” without cheating (e.g., does not peek when counting)” and ”Waits to pay for items without complaint”. A higher score indicates better executive functioning. The Cronbach’s alpha was 0.96 for mothers and 0.95 for partners.

As some partner reports were missing (*n* = 14 for the BRIEF-P and *n* = 15 for the REEF), and they correlated significantly with maternal reports (*r* = 0.51 for the BRIEF-P and *r* = 0.30 for the REEF), maternal reports were used in the main analyses. Partner reports of the BRIEF-P and the REEF were used as sensitivity measures.

### 2.5. Inhibitory Control Tasks

Behavioural tasks were chosen according to five categories of inhibitory control classified by Anderson and Reidy (2012) [49]: motor inhibition (i.e., inhibit motor behaviour at specific moments after learning it), verbal inhibition (i.e., inhibit verbal responses), impulse control (i.e., inhibit an instinctive response), delay of gratification (i.e., resist direct temptation to receive a larger reward after the delay) and go/no-go (i.e., perform certain behaviour after being shown a stimulus and to inhibit that behaviour after being shown a different stimulus). A higher score on the tasks indicates better inhibitory control.

The Flanker task [50] was used to measure motor inhibition. Children were asked to point in the same direction as where a centrally located target fish was swimming towards, ignoring the presence of interfering stimuli (flanking fish oriented in the same or opposite directions). Children who passed the four practice trials were presented with another ten trials, of which three were incongruent trials. Accuracy of the incongruent trials was scored between 0 and 3 (0 = pointing in the wrong direction; 1 = first pointing in the correct direction, then pointing wrongly; 2 = first pointing in the wrong direction, then pointing correctly; 3 = pointing in the correct direction), and subsequently averaged. Forty-nine out of sixty-three children passed the practice trial.

The Whisper task [51,52] was used to measure verbal inhibition. Children who passed the two practice trials were asked to whisper the names of another twelve animal pictures. Answers were coded 0 to 2 for every picture (0 = shout; 1 = normal or mixed tone; 2 = whisper) and averaged. All children passed the practice trial.

The Gift Wrap task [51,52] was used to measure motor inhibition. Before the gift in front of them was wrapped, the children were asked to cover their eyes with their hands and not peek. Wrapping lasted for one minute. Children’s waiting behaviour was coded every five seconds with a score ranging from 0 to 3 (0 = watches wrapping/gift; 1 = peeks; 2 = looks away from wrapping/gift; 3 = closed eyes and/or hands in front of the eyes) and averaged. One child did not understand the task and was therefore excluded from the analysis.

The Gift Delay task [51] was used to measure impulse control. Children were asked to refrain from touching and unwrapping the present placed in front of the child when the examiner left the room for one-and-a-half minutes. Impulse control was measured as latency (measured in seconds) until touching the present.

Due to insufficient variation and low number of children that passed the practice trials in the Snack Delay task (to measure delay of gratification) [51,52] and the Bear Dragon task (to measure go/no-go) [51,53], respectively, these tasks were excluded from the analyses [43].

### 2.6. Scoring of Inhibitory Control Tasks

Video recordings of the inhibitory control tasks were observed and scored independently by two observers. The first five recordings were scored by both observers independently and checked for agreement. Disagreements were discussed and adjusted in the scoring book. Thereafter, the observers only discussed recordings in case of uncertainties. Thirty out of sixty-three recordings were scored by both observers to determine inter-rater reliability. Reliability was quantified by the Intraclass Correlation Coefficient (ICC) relying on absolute agreement. The ICCs for the inhibitory control tasks were good: 0.95 for the Flanker, 0.86 for the Whisper, 0.88 for the Gift wrap, and 0.84 for the Gift delay. Because the tasks measured different forms of inhibitory control as part of the same overarching construct, “lumping” was preferred over “splitting”. Following Willemsen et al. [43], a composite score was created for the inhibitory control tasks by averaging the z-scores. Note that a latent variable could not be created due to violations of the assumptions [43].

### 2.7. Confounders

**Figure 2:**
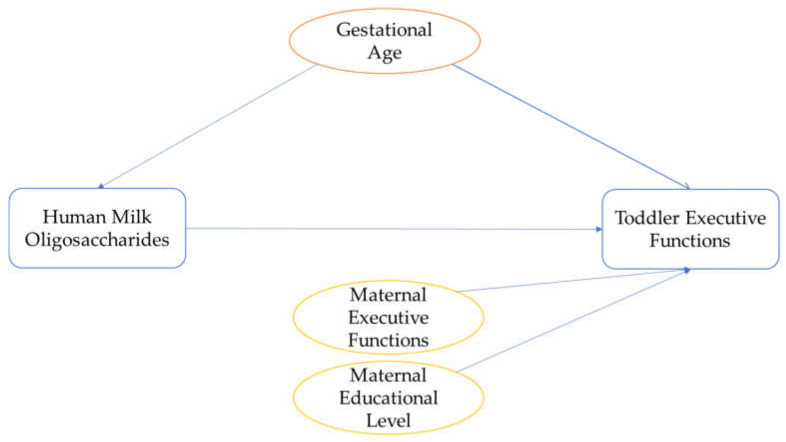
Directed Acyclic Graph for determining confounders. Blue colours represent the predictor and outcome variables. Orange represents the potential confounder related to both the predictor and the outcome, and yellow represents the potential confounder related to the outcome variable.

Potential confounding variables were based on previous literature and plotted in directed acyclic graphs (for DAGs, see Figure 2) [54] to determine their inclusion in the main analysis. Based on the DAGs, the following confounding variables were considered for the main analyses: gestational age at birth [55,56], maternal educational level (ranging from 1, primary education, to 8, university education) [57,58], and executive functioning of the parent(s) [58]. For this last confounder, parental executive functioning was assessed with the Behavior Rating Inventory of Executive Function-Adult (BRIEF-A) [59]. The BRIEF-A is a 75-item self-report questionnaire of executive functioning in adults, scored on a 3-point scale. We reverse-coded the BRIEF-A outcome for interpretation purposes so that higher scores indicate better executive functioning. The Cronbach’s alphas were good for mothers (α = 0.96), and partners (α = 0.93). Similar to the BRIEF-P, some partner reports were missing (*n* = 20), and they correlated significantly with maternal reports (*r* = 0.54). Therefore, only maternal reports were used as confounders, and partner reports of the BRIEF-A were used as sensitivity measures. These potential confounders were correlated with the executive functions and inhibitory control variables to determine their inclusion as confounders in the analyses. Other potential confounders that were considered but eventually excluded based on the DAGs were: maternal age [55], secretor status [18], mode of delivery [60,61], and parity [60]. While low infant birthweight has been associated with HMO composition and cognitive functioning [62,63], the current study only included infants with healthy birthweight; therefore, infant birthweight was not included as confounder. Moreover, breastfeeding duration had previously been assessed in the same cohort with the same outcome variables (i.e., BRIEF-P, REEF, Whisper, Flanker, Gift Wrap, and Gift Delay), and no significant associations were found between breastfeeding duration and these outcomes [43]. Hence, breastfeeding duration was not included as confounder. The following potential confounders were not considered in the DAGs, as no data had been collected: gestational weight gain and maternal body mass index [55].

### 2.8. Missing Data

Excluding the sum variables derived from the original data (e.g., grouped fucosylated HMOs are the sum of specific individual HMOs), 9% of the data were missing. The following milk data were missing: one sample at two weeks and one sample at six weeks. Additional missing milk data were due to some mothers not breastfeeding and therefore unable to provide a sample (two samples at two weeks, four samples at six weeks, and nine samples at twelve weeks). The following questionnaire data were missing: for mothers, REEF (*n* = 1) and BRIEF-A (*n* = 1), and for partners, BRIEF-P (*n* = 14), REEF (*n* = 15), and BRIEF-A (*n* = 20) (of which 14 of each questionnaire were missing because these partners did not join this study at baseline). The following behavioural data were missing: Whisper (*n* = 4), Gift Wrap (*n* = 4), Gift Delay (*n* = 4), and Flanker (*n* = 18, of which 15 were missing because children did not pass the practice trial). The LittleMCAR test from the ‘BaylorEdPsych’ package indicated that data were missing completely at random (X^2^ = 153.173, *p* = 0.549). Missing data were imputed by means of multiple imputation according to Buuren [64] using the ‘mice’ package. Data were imputed 20 times and analyses were thus run 20 times. Results of these analyses were pooled using the *pool* function of the ‘mice’ package, which averages the estimates of the 20 analyses.

### 2.9. Statistical Analyses

All analyses were performed in R version 4.0.2. A 95% confidence interval that doesn’t contain a 0 and a *p*-value of <0.05 were considered statistically significant. Variables were checked for normality, and the following were not normally distributed: 2′FL (at 2, 6, and 12 weeks), 3′SL (at 2, 6, and 12 weeks), 6′SL (at 2, 6, and 12 weeks), grouped fucosylated HMOs (at 6 and 12 weeks), grouped sialylated HMOs (at 2, 6, and 12 weeks), inhibitory control composite score, gestational age, partner executive functions, and maternal educational level. Pearson’s and Spearman’s correlations were performed to correlate normally and non-normally distributed variables, respectively. Furthermore, the area under the curve (AUC) with respect to the ground was calculated for the HMOs of the three milk assessment time points to create one variable that reflects infant exposure to HMOs during the first twelve postnatal weeks [65]. Next, the data used for the final analyses were inspected for outliers. The following variables contained outliers: the AUC of 3′SL (two outliers), the AUC of 6′SL (two outliers), the BRIEF-P filled in by the mother (one outlier), and the inhibitory control composite score (one outlier). The outliers were winsorized [66]. Results of the analyses were similar with and without winsorizing (i.e., including the outliers).

For the main analyses, multiple regression analyses were performed to assess the association between the HMOs (i.e., 2′FL, 3′SL, 6′SL, grouped fucosylated, and grouped sialylated HMOs) and the outcome variables (i.e., executive functions as assessed by the BRIEF-P, REEF, and the inhibitory control composite score). Six models were run, two per outcome variable. The three separate HMOs (2′FL, 3′SL, and 6′SL) were added to three models as predictors of the outcome variables. Because the two HMO groups (fucosylated and sialylated HMOs) are partly derived from the separate HMOs in the first three models, we analysed the HMOs separately. The two HMO groups were added to the three models as predictors for the three outcome variables. These six models were run twice, once including data from exclusively breastfed infants, and once including data from partially breastfed infants.

Sample size could not be adjusted due to the longitudinal nature of our study. We determined the power of our analyses depending on the effect size and sample size, using G*Power (version 3.1). According to Cohen’s [67] guidelines for multiple regression analyses, f^2^  ≥  0.02, f^2^  ≥  0.15, and f^2^  ≥  0.35 represent small, medium, and large effect sizes, respectively. We entered an alpha error probability of 0.05, and six predictors (i.e., the three individual HMOs and the three confounders) for the model with individual HMOs. Five predictors were entered for the model with grouped HMOs. When including exclusively breastfed infants only (*n* = 45), our power is 0.15, 0.72, and 0.97 for detecting small, medium, and large effect sizes, respectively. When including partially breastfed infants (*n* = 63), our power is 0.20, 0.86, and >0.99, for detecting small, medium, and large effect sizes, respectively.

Log-likelihood tests were performed to check which model fit the data best. Models including the confounders fit significantly better than models without confounders. Hence, only results from the models with the confounders were interpreted for the results.

### 2.10. Exploratory Analyses

#### 2.10.1. Clinically Relevant Executive Function Problems 

Multivariate logistic regression analysis was performed to assess the differences between the group of children who scored above the (sub)clinical cut-off of the BRIEF-P (i.e., indicating that these children experience clinically relevant executive function problems), and a group of children without such problems [47]. Seventeen participants scored above the subclinical cut-off of the BRIEF-P (i.e., a t-score of 60 or higher). For the high-low comparison analyses, a contrast group was made by taking the 19 participants who scored the best on executive functions of the BRIEF-P (i.e., t-score of 48 or lower). The dummy outcome variable was being in the group with low executive functions (0) or in the group with high executive functions (1). The predictors were the AUCs of the separate HMOs (2′FL, 3′SL, and 6′SL). The same analyses were performed with the AUCs of the HMO groups as predictors (fucosylated and sialylated HMOs). Note that the BRIEF-P was the only outcome measure in which clinical cut-off values are available.

#### 2.10.2. Individual HMOs and Individual Time Points 

To investigate the effects of other HMOs on executive functions and inhibitory control, we added all HMOs from all time points to a random forest model. In total, 24 HMOs (2′FL, 3-FL, 6′SL, 3′SL, DF-TF-LNH, DFL, four different isomers of F-LNH, IF-LNH-I, LNDFH-I, LNDFH-II, LNFP-I, LNFP-II, LNFP-III, LNFP-V, LNH, LNnH, pLNH, LNT and LNnT combined, LST a, LST b, and LST c) were added to a Random Forest model to assess which HMO had the highest predictive value for the BRIEF-P, the REEF, and the inhibitory control composite. We ran the same analyses for the five HMO groups (grouped fucosylated HMOs, grouped sialylated HMOs, grouped non-fucosylated and grouped non-sialylated HMOs, grouped mono fucosylated HMOs, and grouped Di- and Tri-fucosylated HMOs). One random forest model was run per outcome variable. Thus, three models were run for the exploration of separate HMOs and three models were run for the exploration of HMO groups. The ‘randomForest’ package was used to run the Random forest analyses. We fitted random forest models using the ‘Tuneranger’ package [68]. After that, the HMOs and HMO groups from all time points were added separately to a Random Forest model to assess which HMO at what time point had the most predictive value for the BRIEF-P, the REEF, and the inhibitory control composite (e.g., all HMOs at age 2 weeks were added in one model with the BRIEF-P).

## 3. Results

### 3.1. Descriptives of Study Population Characteristics and Study Variables

**Figure 3:**
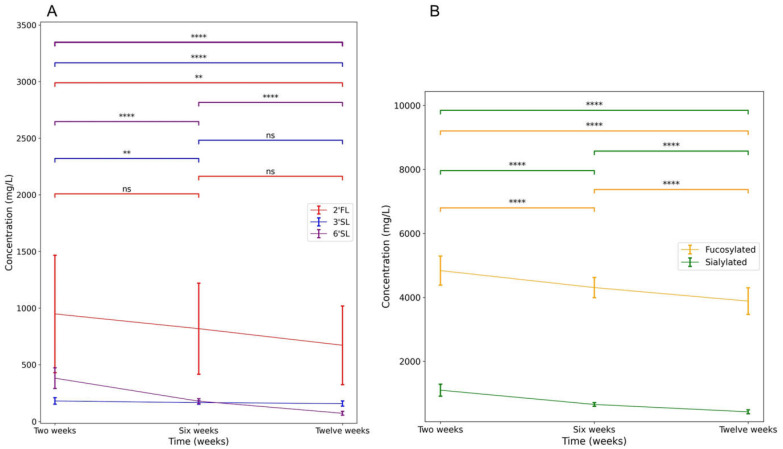
Change in HMO concentrations over the first three months. (**A**) shows the change of individual HMO concentrations. (**B**) shows the change of grouped HMO concentrations. Error bars represent 1 SD of the mean. ns: non-significant; ** *p* < 0.01, **** *p* < 0.0001.

Table 1 shows the descriptive statistics of the study population. Table 2 shows the descriptives of the measured variables including the percentages of exclusively breastfeeding mothers, the measured concentrations of the main HMOs and HMO groups of interest, and the scores on executive functions and inhibitory control tasks. Differences in concentrations over time were tested with a One-Way ANOVA test. Figure 3 shows the significant changes in HMO concentration over time. Concentrations of 6′SL, grouped fucosylated HMOs, and grouped sialylated HMOs decreased significantly over time. Concentrations of 2′FL significantly differed between two weeks and twelve weeks, but not for six weeks. Concentrations of 3′SL at two weeks significantly differed from concentrations at six and twelve weeks. However, concentrations of 3′SL at six weeks did not differ from concentrations at twelve weeks. After adjustment for estimated daily intake, 6′SL and grouped sialylated HMOs decreased significantly over time. Estimated intake of 2′FL, 3′SL, and grouped fucosylated HMOs did not change significantly over time. Scores on the BRIEF-P, the REEF, and the BRIEF-A did not differ significantly between the mother and partner.

**Table 1:** Study population characteristics.

Characteristics	%	*n*
Child sex		
Girl	49.2	31
Boy	50.8	32
Maternal educational level		
Low	0	0
Middle	14.5	9
High	85.5	53
Missing	1.6	1
	Age (±SD)	*n*
Gestational age (weeks)	39.8 (±1.6)	63
Child age (months)	37.6 (±1.1)	63
Maternal age (years) ^1^	34.5 (±3.6)	63
Partner age (years) ^1^	35.9 (±4.1)	47

^1^ Ages at child age three years (date of the home visit).

**Table 2:** Descriptive statistics of measured variables: breastfeeding, HMO levels, executive functions, and inhibitory control.

Breastfeeding	%	*n*		
Exclusive breastfeeding (2 weeks)	86	54		
Exclusive breastfeeding (6 weeks)	89	56		
Exclusive breastfeeding (12 weeks)	78	49		
**HMO levels**	Mean concentration (g/L) (±SD) ^1^	*n*	Estimated daily intake for exclusively breastfed infants (g) (±SD) ^2^	*n*
2′FL 2 weeks	0.95 (±0.52) ^a^	60	0.45 (±0.24)	43
2′FL 6 weeks	0.82 (±0.41) ^ab^	58	0.47 (±0.24)	44
2′FL 12 weeks	0.67 (±0.35) ^b^	54	0.41 (±0.23)	45
3′SL 2 weeks	0.18 (±0.03) ^a^	60	0.08 (±0.01)	43
3′SL 6 weeks	0.17 (±0.01) ^b^	58	0.10 (±0.01)	44
3′SL 12 weeks	0.16 (±0.02) ^b^	54	0.10 (±0.01)	45
6′SL 2 weeks	0.38 (±0.09) ^a^	60	0.18 (±0.04)	43
6′SL 6 weeks	0.18 (±0.02) ^b^	58	0.11 (±0.01)	44
6′SL 12 weeks	0.07 (±0.02) ^c^	54	0.04 (±0.01)	45
Fucosylated HMOs 2 weeks	4.84 (±0.46) ^a^	60	2.31 (±0.18)	43
Fucosylated HMOs 6 weeks	4.31 (±0.32) ^b^	58	2.49 (±0.19)	44
Fucosylated HMOs 12 weeks	3.88 (±0.42) ^c^	54	2.45 (±0.29)	45
Sialylated HMOs 2 weeks	1.10 (±0.19) ^a^	60	0.52 (±0.07)	43
Sialylated HMOs 6 weeks	0.66 (±0.06) ^b^	57	0.38 (±0.04)	44
Sialylated HMOs 12 weeks	0.43 (±0.06) ^c^	54	0.27 (±0.03)	45
**Behaviour**	Score (±SD)	*n*		
**Executive functions questionnaires**				
BRIEF-P mother	95.0 (±15.8)	63		
BRIEF-P partner	97.4 (±18.1)	49		
REEF mother	146.4 (±32.6)	62		
REEF partner	144.9 (±28.0)	48		
BRIEF-A mother	108.2 (±19.7)	62		
BRIEF-A partner	108.3 (±16.0)	43		
**Inhibitory control tasks**				
Flanker	1.3 (±0.7)	45		
Whisper	1.8 (±0.3)	59		
Gift Wrap	2.1 (±0.9)	59		
Gift Delay (seconds)	77.0 (±28.2)	59		

^1^ Adjusted for sample-to-sample variability (with probabilistic quotient normalization). ^2^ Adjusted for daily intake volumes of 480 g, 580 g, and 630 g at weeks two, six, and twelve, respectively, based on previous literature [44]. ^a,b,c^ indicate significant differences (*p* < 0.05) between time points (i.e., ^a^ differs from ^b^ and ^c^, but not from ^a^).

### 3.2. Correlations

#### 3.2.1. Correlations between Executive Function and Inhibitory Control Measures

Correlations between executive function questionnaires and inhibitory control tasks are shown in Table 3. The BRIEF-P and the REEF correlated significantly for mothers (*r* = 0.38), but not for partners. Both the BRIEF-P and the REEF correlated between the mother and partner (*r* = 0.51 and *r* = 0.30, respectively). The BRIEF-A (reflecting parent’s executive functions) and the BRIEF-P (reflecting a toddler’s executive functions) correlated significantly for the mother and partner (*r* = 0.34 and *r* = 0.50, respectively). In addition, the inhibitory control tasks did not intercorrelate. Better performance on the Gift Wrap and the Gift Delay correlated positively with better executive functions as measured by the REEF filled in by the mother (*r* = 0.29 and *r* = 0.37, respectively).

#### 3.2.2. Correlations between Main HMOs of Interest and Behavioural Measures

Next, correlations between the concentrations of HMOs of main interest are shown in Table 4. Concentrations of 2′FL correlated significantly between two and twelve weeks (*r* = 0.30). All concentrations of 2′FL correlated significantly with concentrations of grouped fucosylated HMOs at all time points (*r* ranging from 0.29 to 0.85), except for 2′FL at six weeks and grouped fucosylated HMOs at twelve weeks. Furthermore, 3′SL, 6′SL, and grouped sialylated HMOs correlated negatively over time (*r* ranging from −0.27 to −0.64). After the removal of outliers in these measures, the correlations remained mostly similar.

Correlations between the predictor and outcome variables used in the main models (i.e., AUC of the HMOs, maternal reports of executive functions, and inhibitory control composite) are shown in Table 5. Only the AUC of grouped sialylated HMOs was negatively correlated with the BRIEF-P (*r* = −0.31). No other HMOs were significantly correlated with the executive function and inhibitory control measures.

#### 3.2.3. Correlations between Potential Confounding Variables and Executive Functions Measures

Potential confounding variables were determined beforehand by the use of DAGs (as mentioned in the confounder section) and subsequently correlated with the outcome variables (see Table 6). Only the BRIEF-A correlated significantly with the BRIEF-P (*r* = 0.30) and the inhibitory control composite score (*r* = 0.32). Hence, gestational age and maternal educational level were excluded from the main analysis, and the BRIEF-A was used as a confounding factor for the analyses with the BRIEF-P and the inhibitory control composite score.

### 3.3. Main Analyses

#### 3.3.1. Analyses with Exclusively Breastfed Infants Only

Table 7 shows an overview of the multiple regression analyses, as performed in the exclusively breastfed group. Better executive functioning, as measured with the REEF, was associated with more 2’FL (β: 5.21, 95%CI: 0.84–9.57) and grouped fucosylated HMOs (β: 3.43, 95%CI: 0.30–6.56). These results indicate that higher consumption of human milk concentrations of 2′FL and grouped fucosylated HMOs during infancy are associated with higher executive functions at age three years. No other significant associations were found for the BRIEF-P, the REEF, and the inhibitory control composite. Results were no different with and without winsorizing.

#### 3.3.2. Analyses Including Partially Breastfed Infants

The same analyses were also performed including data from partially breastfed infants (see Supplementary Table S1). The positive association between 2′FL and the REEF found in the exclusively breastfed group was now marginally significant (*p* = 0.06). Additionally, higher levels of sialylated HMOs were associated with worse executive functions, as measured with the BRIEF-P. No other significant results were found, and results were no different with and without winsorizing.

### 3.4. Exploratory Analyses

#### 3.4.1. Clinically Relevant Executive Function Problems

Multiple logistic regression analyses were performed to check the differences between children with high and low executive functions (Table 8). No significant results were found. Results were the same with and without winsorizing. The results including partially breastfed infants were also non-significant (see Supplementary Table S2).

#### 3.4.2. Individual HMOs and Individual Time Points

All HMOs were added to a random forest model using the data of the children that had been exclusively breastfed during the milk sampling period. All models with the BRIEF-P and REEF yielded a high Mean of Squared Residuals (MSR) (ranging from 163 to 212 for the BRIEF-P and from 1025 to 1371 for the REEF) and a negative % variance explained, also after tuning the models. While the models for the inhibitory control composite yielded a low MSR, these models also explained negative variance. Because the model fits for all random forest models could not be improved, indicating that the HMOs we selected were unsuitable for predicting our outcomes, the results of the random forest models were not interpreted. Similar results were found after including data from partially breastfed infants.

To still be able to exploratorily inspect the HMOs at separate time points, we ran multiple regression analyses with the separate HMOs predicting the outcome measures, and corrected for multiple testing by dividing the alpha by the number of predictors in the model [69]. The HMOs at separate time points were not able to significantly predict the outcomes. These results were identical after including the partially breastfed infants.

## 4. Discussion

The goal of this study was to investigate links between human milk HMO concentrations during the first twelve postpartum weeks, and executive functions and inhibitory control at three years of age. The analyses performed in the group of exclusively breastfed infants during the 12-week milk sampling period provided evidence that higher milk concentrations of 2′FL and grouped fucosylated HMOs during the first twelve postnatal weeks were associated with better executive functions at age three, as measured with the REEF questionnaire. When partially breastfed infants were added to the analyses, similar results for 2′FL were produced and a negative association between grouped sialylated HMOs and executive functions, as measured with the BRIEF-P questionnaire, appeared. No associations were found with 3′SL, 6′SL, and the inhibitory control composite score. The results from our random forest models with HMOs measured at single time points could not be interpreted due to poor model fits.

We found evidence for an association between higher levels of 2′FL in the first twelve weeks and better executive functions at age three years. This finding seems robust as it appeared in the analyses with and without including partially breastfed infants. Results of animal studies are also in line with 2′FL leading to better cognition (for a review, see Docq et al. [25]). Early life administration of 2′FL enhanced long-term potentiation (LTP, involved in memory and learning) in rats, improved recognition memory in pigs, and improved performance in operant learning paradigms in mice [26,31,70]. Moreover, our results are consistent with two human studies that found an association between 2′FL at one month and better motoric development at six months [9], and between 2′FL at one month and better cognition at twenty-four months [10]. Interestingly, Berger et al. [10] also measured 2′FL concentrations at six months and did not find an association with cognition. Likewise, Jorgensen et al. [12] also did not find a link between 2′FL at six months and child cognition or executive functions. As such, it might be speculated from these and our findings that early life exposure to 2′FL might be especially important for later cognitive development. It should also be noted that some human studies did not find an association between 2′FL and better cognitive outcomes. Cho et al. [11] found no evidence for a link between 2′FL concentration (measured at different times for individual infants between ages 2 to 25 months) and cognition (assessed at ages between 2 to 25 months). A potential underlying mechanism associating 2′FL with later cognition is the gut microbiota. Indeed, Vazquez et al. found that 2′FL ingestion in rodents improved learning ability and LTP enhancement [33], but only when the connection of the vagus nerve was intact [70]. Ingestion of 2′FL may have stimulated the production of low molecular components by the gut microbiota, possibly improving executive functions. Conversely, the gut bacteria can alter the integrity of 2′FL [71], causing 2′FL to reach the brain in a different form. Different forms of 2′FL can exert different effects on LTP in the brain [70]. In addition, fucosyllactose is utilised by *Bifidobacteria* to promote their growth, which may result in positive effects on the brain [72,73,74]. For future studies, it is therefore suggested to include the gut microbiota when investigating the role of HMOs on cognitive outcomes. Additionally, because HMO levels decrease over time, and both Jorgensen et al. [12] and Berger et al. [10] found no evidence for a relation between future cognition and 2′FL at six months, future sufficiently powered human studies should consider multiple milk samples over a longer period of time to identify sensitive periods for 2′FL concentrations to impact the developing brain.

Our findings also showed that higher concentrations of grouped fucosylated HMOs were present in the human milk of children with higher levels of executive functions. Jorgensen et al. [12] found a positive link between grouped fucosylated HMOs at six months and language, but not executive functions, at age 18 months. Moreover, as more human studies investigating fucosylated HMOs as a group are lacking, and animal studies on grouped fucosylated HMOs and cognition are scarce, we can only cautiously speculate that grouped fucosylated HMOs may exert positive effects on cognition. Most HMO research to date focused on specific, individual fucosylated HMOs, including 2′FL. For this reason and given our positive findings, future studies may consider also investigating fucosylated HMOs as a group, next to individual HMOs, as the structure of fucosylated HMOs indicate that their physiological functions may be similar [14]. More mechanistic studies are also necessary to investigate how grouped fucosylated HMOs might improve cognitive outcomes.

Contrary to our hypothesis, and only in the analyses with the partially breastfed infants included, higher concentrations of grouped sialylated HMOs in mother’s milk predicted worse executive functions in 3-year-old children, as measured with the BRIEF-P. Only Jorgensen et al. [12] investigated grouped sialylated HMOs in humans and found higher levels of grouped sialylated HMOs to be associated with improved language performance at 18 months. Note that our positive associations between 2′FL and grouped fucosylated HMOs and executive functions were obtained with the REEF questionnaire. Hence, these apparent discrepancies in our results might be explained by the fact that the BRIEF-P assesses child executive functions more in general, while the REEF assesses child behaviour in specific everyday situations. The design of these questionnaires may also explain why paternal BRIEF-P and REEF did not correlate, as in traditional households (like often is the case in the Netherlands [75]), fathers, compared to mothers, spend less time with their children. Fathers may thus have a better view of their child’s general executive functions as compared to their child’s executive functions in specific daily situations. This could explain why the BRIEF-P was more strongly correlated between parents, compared to the REEF. The BRIEF-P may therefore be a more robust measure of executive functions in general, while the REEF might be more suitable for caregivers who spend more time with their children in different situations. Although the BRIEF-P has been used more often, the use of the newer REEF has been rising.

Our results on grouped sialylated HMOs and worse executive functions were only found when partially breastfed infants were included in the analyses. Because partially breastfed infants by definition consume fewer HMOs than exclusively breastfed infants, we cannot exclude the possibility that these associations between grouped sialylated HMOs and worse executive functions that were only found in the partially breastfed infants may be a chance finding. Additionally, our main analyses were performed on the exclusively breastfed group to correct for potential noise that formula feeding may cause. Some formula feeding includes galactooligosaccharides (GOS) and fructooligosaccharides (FOS) which mimic the effects that HMOs have on gut bacteria [76,77], and hence potentially on the brain [21,22,23]. For this reason, and because the findings differed for the exclusively breastfed versus any breastfed group, we refrain from further interpreting these results.

Furthermore, we found no evidence for a relation between 3′SL and 6′SL concentrations and executive functions. Previous results on these HMOs are mixed. Two human studies and one animal study found a positive association with 3′SL and better future cognitive outcomes [11,12,78], while one human and one animal study found no evidence for an association between 3′SL and future cognition [10,79]. Regarding 6′SL, one human study found an association between higher concentrations of 6′SL at one month and better cognition [9], while another found an association between higher concentrations of 6′SL and a smaller change in infant head circumference between 6–18 months [12]. Finally, two studies found no evidence for a link between 6′SL and cognition at age 24 months [10,11]. In piglets, ingestion of 3′SL and 6′SL are related to an increase in sialic acid concentration in the cerebellum and the hippocampus, as well as an expanded hippocampus [80,81]. Whether this mechanism is associated with better executive functions is still unclear. Nonetheless, it is premature to draw conclusions regarding individual sialylated HMOs, as results in human studies are inconsistent, likely due to the different methodologies used and ages assessed. Sufficiently powered replication studies are necessary to obtain clarity on if, how, and when sialylated HMOs are associated with child cognition. Curiously, the correlations between all sialylated HMOs (including grouped sialylated HMOs) were negative over time, meaning that higher levels of sialylated HMOs at one point were correlated with lower levels of sialylated HMOs at another time point. This finding was robust, since the removal of outliers did not change these correlations. It is difficult to speculate why these correlations are negative over the first 12 postnatal weeks. How sialylated HMOs develop over time thus requires more research. Future studies on this topic may benefit from adding different time-variant factors, such as maternal diet, or maternal condition and recovery after delivery [55].

Our study has several strengths. To our knowledge, this study is the first to assess HMO concentrations at three time points early in life and relate these concentrations to cognitive outcomes in toddlerhood. The multiple time points allowed us to investigate HMO concentrations during a critical and sensitive period in life [82]. Second, we used two different types of questionnaires, filled in by mothers and their partners, and several behavioural tasks to provide a more robust view of child executive functions. A good addition to these measures would be to use eye-tracking [83] or MRI scans [84] for more fine-grained assessments [1]. Our study also has its limitations. The individual milk volume consumption was not measured. This resulted in our estimating HMO exposure based on mean daily intakes known from the literature which is less accurate. Although tedious, future research may benefit from instructing mothers to weigh their infants before and after each feeding to obtain a more precise estimate of their daily milk consumption [85,86]. Next, the generalizability of our results is limited by our mostly highly educated sample. Lastly, our relatively small sample size reduced our statistical power. However, we preserved our power as much as possible by reducing the number of statistical tests performed, calculating the AUC of the three HMO measures, creating a composite score of the observed inhibitory control test scores, and using partner scores as sensitivity measures.

## 5. Conclusions

Despite knowing the beneficial effects of human milk, it is currently one of the most under-investigated biological systems in life sciences [1]. Specifically, human studies investigating HMOs in relation to cognitive outcomes in early childhood are scarce. We found evidence for an association between 2′FL and grouped fucosylated HMOs during the first twelve postnatal weeks and better child executive functions at age three. In the future, larger replication studies should consider collecting multiple mothers’ milk samples in early life and extending these findings to later ages as well. Additionally, studies may benefit from including the gut microbiota in their analyses to be able to investigate the mechanisms underlying HMO associations with child neurodevelopment. Studies should also investigate the effects of HMOs on the development of vulnerable groups who require tailored nutrition but do not always have access to human milk (e.g., preterm born infants). Such studies would aid in the determination of sensitive periods in which HMOs may exert the largest positive effects on cognition and executive functions.

## Figures and Tables

**Table 3:** Correlations between executive function measures and inhibitory control measures.

	BRIEF-P Mother	BRIEF-P Partner	REEF Mother	REEF Partner	BRIEF-A Mother	BRIEF-A Partner	Flanker	Whisper	Gift Wrap	Gift Delay
**BRIEF-P Mother**	-									
**BRIEF-P Partner**	0.51 ***	-								
**REEF Mother**	0.38 **	0.23	-							
**REEF Partner**	0.03	0.08	0.30 *	-						
**BRIEF-A Mother**	0.34 **	0.26	−0.14	−0.06	-					
**BRIEF-A Partner**	0.34 *	0.50 **	0.13	0.05	0.54 ***	-				
**Flanker**	0.07	0.23	0.15	0.23	−0.02	0.25	-			
**Whisper**	0.00	−0.17	0.00	0.04	−0.06	−0.21	0.04	-		
**Gift Wrap**	−0.03	−0.02	0.29 *	0.18	−0.24	−0.02	0.10	−0.07	-	
**Gift Delay**	0.10	0.00	0.37 **	0.15	−0.32 *	−0.23	0.19	0.12	0.21	-

Note: BRIEF-P and BRIEF-A are reverse-scored (i.e., higher scores indicate better executive functions). * *p* < 0.05. ** *p* < 0.01. *** *p* < 0.001. *n* = 63.

**Table 4:** Correlations between concentrations of individual HMOs and grouped HMOs.

	2′FL (2 w)	2′FL (6 w)	2′FL (12 w)	3′SL (2 w)	3′SL (6 w)	3′SL (12 w)	6′SL (2 w)	6′SL (6 w)	6′SL (12 w)	Fuc HMOs (2 w)	Fuc HMOs (6 w)	Fuc HMOs (12 w)	Sial HMOs (2 w)	Sial HMOs (6 w)	Sial HMOs (12 w)
2′FL (2 w)	-														
2′FL (6 w)	0.26														
2′FL (12 w)	0.30 *	0.14													
3′SL (2 w)	0.08	−0.08	−0.17												
3′SL (6 w)	0.02	0.25	−0.24	−0.30 *											
3′SL (12 w)	0.08	−0.07	0.37 **	−0.64 ***	−0.42 **										
6′SL (2 w)	0.19	−0.12	−0.03	0.08	−0.04	−0.07									
6′SL (6 w)	−0.13	0.18	−0.13	−0.17	−0.03	0.14	−0.39 **								
6′SL (12 w)	−0.12	−0.09	0.17	−0.10	0.24	−0.03	−0.64 ***	−0.27 *							
Fuc HMOs (2 w)	0.66 ***	0.43 **	0.34 *	0.28 *	−0.03	−0.10	0.06	−0.10	0.00						
Fuc HMOs (6 w)	0.29 *	0.85 ***	0.30 *	−0.20	0.42 **	−0.08	−0.03	0.20	−0.19	0.33 *					
Fuc HMOs (12 w)	0.34 *	0.01	0.73 ***	−0.21	−0.25	0.40 **	−0.01	−0.15	0.15	0.14	0.05				
Sial HMOs (2 w)	0.25	−0.09	−0.05	0.41 **	−0.03	−0.29 *	0.71 ***	−0.30 *	−0.51 ***	0.39 **	−0.06	−0.04			
Sial HMOs (6 w)	−0.37 **	0.22	−0.20	−0.29 *	0.47 ***	−0.13	−0.24	0.48 ***	−0.10	−0.45 **	0.41 **	−0.38 **	−0.43 **		
Sial HMOs (12 w)	0.09	−0.11	0.32 *	−0.46 ***	−0.26	0.77 ***	−0.25	−0.14	0.36 **	−0.10	−0.20	0.47 ***	−0.45 ***	−0.30 *	-

Note: Correlations are denoted as *r*. Fuc: Fucosylated, Sial: Sialylated, 2 w: two weeks, 6 w: six weeks, 12 w: twelve weeks. HMO concentrations are in grams per litre and adjusted for sample-to-sample variability (*n* = 63). * *p* < 0.05. ** *p* < 0.01. *** *p* < 0.001.

**Table 5:** Correlations between maternal reports on executive functions, inhibitory control composite, and the AUCs of the HMOs (adjusted for sample-to-sample variability, percentage breastfeeding, and estimated daily intake).

	BRIEF-P by Mother	REEF by Mother	Inhibitory Control Composite
**AUC of 2’FL**	0.00	0.16	0.04
**AUC of 3’SL**	0.20	−0.12	−0.06
**AUC of 6’SL**	0.14	−0.16	−0.13
**AUC of Fucosylated HMOs**	−0.17	0.21	0.14
**AUC of Sialylated HMOs**	−0.31 *	0.03	−0.09

Notes: Correlations are based on imputed data (*n* = 63). HMOs mentioned in this table are the AUCs of the HMOs in grams consumed at 2, 6, and 12 weeks; the BRIEF-P is reverse-coded to correspond with the other executive functions and inhibition measures (i.e., higher BRIEF-P scores indicate better executive functions). * *p* < 0.05.

**Table 6:** Correlations between executive function measures and potential confounding variables.

	BRIEF-P Mother	REEF Mother	Inhibitory Control Composite	Gestational Age	Mother Educational Level	BRIEF-A Mother
**BRIEF-P Mother**	-					
**REEF Mother**	0.33 **	-				
**Inhibitory control composite**	0.07	0.34 **	-			
**Gestational age**	−0.01	−0.07	−0.05	-		
**Mother educational level**	−0.04	0.18	0.17	−0.02	-	
**BRIEF-A Mother**	0.30 *	−0.07	0.32 *	0.07	0	-

Note: Correlations are based on imputed data (*n* = 63). The BRIEF-P is reverse-coded to correspond with the other executive functions and inhibition measures (i.e., higher BRIEF-P scores indicate better executive functions). * *p* < 0.05, ** *p* < 0.01.

**Table 7:** Associations between HMOs and executive functions (BRIEF-P and REEF) and inhibitory control (exclusively breastfed infants).

Effect	Estimate (95% CI)	Standard Error	*p*-Value
**BRIEF-P Model 1**			
Intercept	147.65 (47.08–248.21) **	49.76	0.005
2′FL	−0.34 (−2.07–1.40)	0.86	0.70
6′SL	−39.22 (−85.79–7.35)	23.04	0.10
3′SL	−29.15 (−118.13–59.83)	44.03	0.51
BRIEF-A	0.15 (−0.048–0.35)	0.10	0.13
**BRIEF-P Model 2**			
Intercept	108.24 (22.69–193.78) *	42.36	0.015
Fucosylated HMOs	−0.18 (−1.45–1.08)	0.63	0.77
Sialylated HMOs	−7.88 (−28.30–12.53)	10.11	0.44
BRIEF-A	0.18 (−0.02–0.38)	0.10	0.08
**REEF Model 1**			
Intercept	264.44 (33.40–495.48) *	114.40	0.03
2′FL	5.21 (0.84–9.57) *	2.16	0.02
6′SL	−14.33 (−131.61–102.96)	58.08	0.81
3′SL	−138.79 (−360.91–83.34)	109.99	0.21
**REEF Model 2**			
Intercept	122.34 (−76.57–321.26)	98.57	0.22
Fucosylated HMOs	3.43 (0.30–6.56) *	1.55	0.03
Sialylated HMOs	−15.75 (−66.45–34.95)	25.12	0.53
**Inhibitory control Model 1**			
Intercept	1.31 (−2.53–5.15)	1.90	0.49
2′FL	0.01 (−0.05–0.08)	0.03	0.70
6′SL	−0.37 (−2.15–1.41)	0.88	0.68
3′SL	0.10 (−3.29–3.50)	1.68	0.95
BRIEF-A	−0.01 (−0.02–−0.002) *	0.004	0.02
**Inhibitory control Model 2**			
Intercept	2.24 (−0.82–5.30)	1.52	0.15
Fucosylated HMOs	0.02 (−0.02–0.07)	0.02	0.27
Sialylated HMOs	−0.47 (−1.20–0.26)	0.36	0.20
BRIEF-A	−0.01 (−0.02–−0.002) *	0.004	0.01

Note that analyses were performed on exclusively breastfed infants only, *n* = 45. The REEF models did not include confounders as none of the potential confounders correlated with the REEF. The BRIEF-P is reverse-coded to correspond with the other executive functions and inhibition measures (i.e., higher BRIEF-P scores indicate better executive functions). All HMOs and HMO groups mentioned in this table are the Area Under the Curve. *: *p* < 0.05, **: *p* < 0.01.

**Table 8:** Multiple logistic regression results of the relation between the HMOs and HMO groups and the BRIEF-P (exclusively breastfed infants).

Effect	Estimate (95% CI)	Standard Error	*p*-Value
**BRIEF-P Model 1**			
**Intercept**	19.90 (−10.91–61.23)	17.45	0.27
**2′FL**	−0.03 (−0.63–0.47)	0.26	0.91
**6′SL**	−10.36 (−26.00–1.86)	6.80	0.15
**3′SL**	−12.84 (−44.72–11.80)	13.73	0.36
**BRIEF-A**	0.03 (−0.02–0.10)	0.03	0.29
**BRIEF-P Model 2**			
**Intercept**	1.68 (−22.06–24.82)	11.53	0.89
**Fucosylated HMOs**	−0.04 (−0.42–0.27)	0.16	0.79
**Sialylated HMOs**	−1.16 (−7.71–5.07)	3.14	0.72
**BRIEF-A**	0.03 (−0.01–0.09)	0.02	0.18

Note that the analyses were performed on exclusively breastfed infants only, *n* = 45. All HMOs and HMO groups mentioned in this table are the Area Under the Curve. BRIEF-P coded as: 1, representing the high executive functions group, and 0, representing the low executive functions group. Hence, positive values indicate a positive association between higher levels of HMOs and high executive functions.

## Data Availability

Data described in the manuscript will not be made publicly available due to the participants not having given permission for this. The data, code book, and analytic code are available upon request to C. de Weerth at Department of Cognitive Neuroscience, Donders Institute for Brain, Cognition and Behaviour, Radboud University Medical Center, Nijmegen, The Netherlands (e-mail: carolina.deweerth@radboudumc.nl).

## Supplementary Materials

The following supporting information can be downloaded at: https://www.mdpi.com/article/10.3390/nu15061463/s1, Table S1: Associations between HMOs and HMO groups and measures of executive functioning including partially breastfed infants; Table S2: Multiple logistic regression results between the HMOs and HMO groups and the BRIEF-P including partially breastfed infants.

## References

[1] De Weerth C., Aatsinki A.-K., Azad M.B., Bartol F.F., Bode L., Collado M.C., Dettmer A.M., Field C.J., Guilfoyle M., Hinde K. (2022). Human Milk: From Complex Tailored Nutrition to Bioactive Impact on Child Cognition and Behavior. Crit. Rev. Food Sci. Nutr..

[2] Victora C.G., Bahl R., Barros A.J.D., França G.V.A., Horton S., Krasevec J., Murch S., Sankar M.J., Walker N., Rollins N.C. (2016). Breastfeeding in the 21st Century: Epidemiology, Mechanisms, and Lifelong Effect. Lancet.

[3] Hou L., Li X., Yan P., Li Y., Wu Y., Yang Q., Shi X., Ge L., Yang K. (2021). Impact of the Duration of Breastfeeding on the Intelligence of Children: A Systematic Review with Network Meta-Analysis. Breastfeed. Med..

[4] Bartol F.F., Wiley A.A., George A.F., Miller D.J., Bagnell C.A. (2017). Physiology and Endocrinology Symposium: Postnatal Reproductive Development and the Lactocrine Hypothesis. J. Anim. Sci..

[5] Liu H., Radlowski E.C., Conrad M.S., Li Y., Dilger R.N., Johnson R.W. (2014). Early Supplementation of Phospholipids and Gangliosides Affects Brain and Cognitive Development in Neonatal Piglets. J. Nutr..

[6] Bartol F.F., Wiley A.A., Bagnell C.A. (2008). Epigenetic Programming of Porcine Endometrial Function and the Lactocrine Hypothesis. Reprod. Domest. Anim..

[7] Bode L. (2012). Human Milk Oligosaccharides: Every Baby Needs a Sugar Mama. Glycobiology.

[8] Wang B. (2012). Molecular Mechanism Underlying Sialic Acid as an Essential Nutrient for Brain Development and Cognition. Adv. Nutr..

[9] Oliveros E., Martín M.J., Torres-Espínola F.J., Segura-Moreno T., Ramírez M., Santos A., Buck R., Rueda R., Escudero M., Catena A. (2021). Human Milk Levels of 2′-Fucosyllactose and 6′-Sialyllactose Are Positively Associated with Infant Neurodevelopment and Are Not Impacted by Maternal BMI or Diabetic Status. J. Nutr. Food Sci..

[10] Berger P.K., Plows J.F., Jones R.B., Alderete T.L., Yonemitsu C., Poulsen M., Ryoo J.H., Peterson B.S., Bode L., Goran M.I. (2020). Human Milk Oligosaccharide 2′-Fucosyllactose Links Feedings at 1 Month to Cognitive Development at 24 Months in Infants of Normal and Overweight Mothers. PLoS ONE.

[11] Cho S., Zhu Z., Li T., Baluyot K., Howell B.R., Hazlett H.C., Elison J.T., Hauser J., Sprenger N., Wu D. (2021). Human Milk 3′-Sialyllactose Is Positively Associated with Language Development during Infancy. Am. J. Clin. Nutr..

[12] Jorgensen J.M., Young R., Ashorn P., Ashorn U., Chaima D., Davis J.C.C., Goonatilleke E., Kumwenda C., Lebrilla C.B., Maleta K. (2021). Associations of Human Milk Oligosaccharides and Bioactive Proteins with Infant Growth and Development among Malawian Mother-Infant Dyads. Am. J. Clin. Nutr..

[13] Tonon K.M., Miranda A., Abrão A.C.F.V., de Morais M.B., Morais T.B. (2019). Validation and Application of a Method for the Simultaneous Absolute Quantification of 16 Neutral and Acidic Human Milk Oligosaccharides by Graphitized Carbon Liquid Chromatography—Electrospray Ionization—Mass Spectrometry. Food Chem..

[14] Bode L., Jantscher-Krenn E. (2012). Structure-Function Relationships of Human Milk Oligosaccharides. Adv. Nutr..

[15] Soyyilmaz B., Mikš M.H., Röhrig C.H., Matwiejuk M., Meszaros-matwiejuk A., Vigsnæs L.K. (2021). The Mean of Milk: A Review of Human Milk Oligosaccharide Concentrations throughout Lactation. Nutrients.

[16] Thum C., Wall C.R., Weiss G.A., Wang W., Szeto I.M.Y., Day L. (2021). Changes in Hmo Concentrations throughout Lactation: Influencing Factors, Health Effects and Opportunities. Nutrients.

[17] Borewicz K., Gu F., Saccenti E., Hechler C., Beijers R., de Weerth C., van Leeuwen S.S., Schols H.A., Smidt H. (2020). The Association between Breastmilk Oligosaccharides and Faecal Microbiota in Healthy Breastfed Infants at Two, Six, and Twelve Weeks of Age. Sci. Rep..

[18] Thurl S., Munzert M., Boehm G., Matthews C., Stahl B. (2017). Systematic Review of the Concentrations of Oligosaccharides in Human Milk. Nutr. Rev..

[19] De Weerth C. (2017). Do Bacteria Shape Our Development? Crosstalk between Intestinal Microbiota and HPA Axis. Neurosci. Biobehav. Rev..

[20] Cryan J.F., O’riordan K.J., Cowan C.S.M., Sandhu K.V., Bastiaanssen T.F.S., Boehme M., Codagnone M.G., Cussotto S., Fulling C., Golubeva A.V. (2019). The Microbiota-Gut-Brain Axis. Physiol. Rev..

[21] Bode L. (2015). The Functional Biology of Human Milk Oligosaccharides. Early Hum. Dev..

[22] Totten S.M., Zivkovic A.M., Wu S., Ngyuen U., Freeman S.L., Ruhaak L.R., Darboe M.K., German J.B., Prentice A.M., Lebrilla C.B. (2012). Comprehensive Profiles of Human Milk Oligosaccharides Yield Highly Sensitive and Specific Markers for Determining Secretor Status in Lactating Mothers. J. Proteome Res..

[23] Underwood M.A., German J.B., Lebrilla C.B., Mills D.A. (2014). Bifidobacterium Longum Subspecies Infantis: Champion Colonizer of the Infant Gut. Pediatr. Res..

[24] Dalile B., Van Oudenhove L., Vervliet B., Verbeke K. (2019). The Role of Short-Chain Fatty Acids in Microbiota–Gut–Brain Communication. Nat. Rev. Gastroenterol. Hepatol..

[25] Docq S., Spoelder M., Wang W., Homberg J.R. (2020). The Protective and Long-Lasting Effects of Human Milk Oligosaccharides on Cognition in Mammals. Nutrients.

[26] Fleming S.A., Mudd A.T., Hauser J., Yan J., Metairon S., Steiner P., Donovan S.M., Dilger R.N. (2020). Dietary Oligofructose Alone or in Combination with 2′-Fucosyllactose Differentially Improves Recognition Memory and Hippocampal MRNA Expression. Nutrients.

[27] Fleming S.A., Mudd A.T., Hauser J., Yan J., Metairon S., Steiner P., Donovan S.M., Dilger R.N. (2020). Human and Bovine Milk Oligosaccharides Elicit Improved Recognition Memory Concurrent with Alterations in Regional Brain Volumes and Hippocampal MRNA Expression. Front. Neurosci..

[28] Obelitz-Ryom K., Bering S.B., Overgaard S.H., Eskildsen S.F., Ringgaard S., Olesen J.L., Skovgaard K., Pankratova S., Wang B., Brunse A. (2019). Bovine Milk Oligosaccharides with Sialyllactose Improves Cognition in Preterm Pigs. Nutrients.

[29] Wang B., Yu B., Karim M., Hu H., Sun Y., McGreevy P., Petocz P., Held S., Brand-Miller J. (2007). Dietary Sialic Acid Supplementation Improves Learning and Memory in Piglets. Am. J. Clin. Nutr..

[30] Oliveros E., Vázquez E., Barranco A., Ramírez M., Gruart A., Delgado-García J.M., Buck R., Rueda R., Martín M.J. (2018). Sialic Acid and Sialylated Oligosaccharide Supplementation during Lactation Improves Learning and Memory in Rats. Nutrients.

[31] Oliveros E., Ramirez M., Vazquez E., Barranco A., Gruart A., Delgado-Garcia J.M., Buck R., Rueda R., Martin M.J. (2016). Oral Supplementation of 2′-Fucosyllactose during Lactation Improves Memory and Learning in Rats. J. Nutr. Biochem..

[32] Berger B., Porta N., Foata F., Grathwohl D., Delley M., Moine D., Charpagne A., Siegwald L., Descombes P., Alliet P. (2020). Linking Human Milk Oligosaccharides, Infant Fecal Community Types, and Later Risk to Require Antibiotics. MBio.

[33] Vázquez E., Barranco A., Ramírez M., Gruart A., Delgado-García J.M., Martínez-Lara E., Blanco S., Martín M.J., Castanys E., Buck R. (2015). Effects of a Human Milk Oligosaccharide, 2′-Fucosyllactose, on Hippocampal Long-Term Potentiation and Learning Capabilities in Rodents. J. Nutr. Biochem..

[34] Krug M., Wagner M., Staak S., Smalla K.H. (1994). Fucose and Fucose-Containing Sugar Epitopes Enhance Hippocampal Long-Term Potentiation in the Freely Moving Rat. Brain Res..

[35] Matthies H., Staak S., Krug M. (1996). Fucose and Fucosyllactose Enhance In-Vitro Hippocampal Long-Term Potentiation. Brain Res..

[36] Hauser J., Pisa E., Arias Vásquez A., Tomasi F., Traversa A., Chiodi V., Martin F.P., Sprenger N., Lukjancenko O., Zollinger A. (2021). Sialylated Human Milk Oligosaccharides Program Cognitive Development through a Non-Genomic Transmission Mode. Mol. Psychiatry.

[37] Wang B. (2009). Sialic Acid Is an Essential Nutrient for Brain Development and Cognition. Annu. Rev. Nutr..

[38] Schnaar R.L., Gerardy-Schahn R., Hildebrandt H. (2014). Sialic Acids in the Brain: Gangliosides and Polysialic Acid in Nervous System Development, Stability, Disease, and Regeneration. Physiol. Rev..

[39] Cook F., Giallo R., Hiscock H., Mensah F., Sanchez K., Reilly S. (2019). Infant Regulation and Child Mental Health Concerns: A Longitudinal Study. Pediatrics.

[40] Montroy J.J., Bowles R.P., Skibbe L.E., McClelland M.M., Morrison F.J. (2016). The Development of Self-Regulation across Early Childhood. Dev. Psychol..

[41] Hechler C., Beijers R., Riksen-Walraven J.M., de Weerth C. (2018). Are Cortisol Concentrations in Human Breast Milk Associated with Infant Crying?. Dev. Psychobiol..

[42] Hechler C., Beijers R., Riksen-Walraven M., de Weerth C. (2019). Prenatal Predictors of Postnatal Quality of Caregiving Behavior in Mothers and Fathers. Parenting.

[43] Willemsen Y., Beijers R., Arias Vasquez A., de Weerth C. (2021). Do Breastfeeding History and Diet Quality Predict Inhibitory Control at Preschool Age?. Nutrients.

[44] Gu F., Kate G.A.T., Arts I.C.W., Penders J., Thijs C., Lindner C., Nauta A., Van Leusen E., Van Leeuwen S.S., Schols H.A. (2021). Combining HPAEC-PAD, PGC-LC-MS, and 1D 1H NMR to Investigate Metabolic Fates of Human Milk Oligosaccharides in 1-Month-Old Infants: A Pilot Study. J. Agric. Food Chem..

[45] Gu F., Wang S., Beijers R., De Weerth C., Schols H.A. (2021). Structure-Specific and Individual-Dependent Metabolization of Human Milk Oligosaccharides in Infants: A Longitudinal Birth Cohort Study. J. Agric. Food Chem..

[46] Institute of Medicine (U.S.) (1992). Subcommittee on Nutrition during Lactation. Nutrition during Lactation.

[47] Sherman E.M.S., Brooks B.L. (2010). Behavior Rating Inventory of Executive Function—Preschool Version (BRIEF-P): Test Review and Clinical Guidelines for Use. Child Neuropsychol..

[48] Nilsen E.S., Huyder V., McAuley T., Liebermann D. (2017). Ratings of Everyday Executive Functioning (REEF): A Parent-Report Measure of Preschoolers’ Executive Functioning Skills. Psychol. Assess..

[49] Anderson P.J., Reidy N. (2012). Assessing Executive Function in Preschoolers. Neuropsychol. Rev..

[50] Eriksen B.A., Eriksen C.W. (1974). Effects of Noise Letters upon the Identification of a Target Letter in a Nonsearch Task. Percept. Psychophys..

[51] Kochanska G., Murray K., Jacques T.Y., Koenig A.L., Vandegeest K.A. (1996). Inhibitory Control in Young Children and Its Role in Emerging Internalization. Child Dev..

[52] Beijers R., Riksen-Walraven M., Putnam S., de Jong M., de Weerth C. (2013). Early Non-Parental Care and Toddler Behaviour Problems: Links with Temperamental Negative Affectivity and Inhibitory Control. Early Child. Res. Q..

[53] Reed M.A., Pien D.L., Rothbart M.K. (1984). Inhibitory Self-Control in Preschool Children. Merrill. Palmer. Q..

[54] Cinelli C., Forney A., Pearl J. (2021). A Crash Course in Good and Bad Controls. Sociol. Methods Res..

[55] Han S.M., Derraik J.G.B., Binia A., Sprenger N., Vickers M.H., Cutfield W.S. (2021). Maternal and Infant Factors Influencing Human Milk Oligosaccharide Composition: Beyond Maternal Genetics. J. Nutr..

[56] Yang S., Platt R.W., Kramer M.S. (2010). Variation in Child Cognitive Ability by Week of Gestation among Healthy Term Births. Am. J. Epidemiol..

[57] Ardila A., Rosselli M., Matute E., Guajardo S. (2005). The Influence of the Parents’ Educational Level on the Development of Executive Functions. Dev. Neuropsychol..

[58] Kao K., Nayak S., Doan S.N., Tarullo A.R. (2018). Relations Between Parent EF and Child EF: The Role of Socioeconomic Status and Parenting on Executive Functioning in Early Childhood. Transl. Issues Psychol. Sci..

[59] Roth R.M., Gioia G.A. (2005). Behavior Rating Inventory of Executive Function—Adult Version. Psychol. Assess. Resour..

[60] Samuel T.M., Binia A., de Castro C.A., Thakkar S.K., Billeaud C., Agosti M., Al-Jashi I., Costeira M.J., Marchini G., Martínez-Costa C. (2019). Impact of Maternal Characteristics on Human Milk Oligosaccharide Composition over the First 4 Months of Lactation in a Cohort of Healthy European Mothers. Sci. Rep..

[61] Ahlqvist V.H., Ekström L.D., Jónsson-Bachmann E., Tynelius P., Madley-Dowd P., Neovius M., Magnusson C., Berglind D. (2022). Caesarean Section and Its Relationship to Offspring General Cognitive Ability: A Registry-Based Cohort Study of Half a Million Young Male Adults. Evid. Based. Ment. Health.

[62] Cheng E.R., Poehlmann-Tynan J., Mullahy J., Witt W.P. (2014). Cumulative Social Risk Exposure, Infant Birthweight, and Cognitive Delay in Infancy. Acad. Pediatr..

[63] Hack M., Klein N.K., Taylor H.G. (1995). Long-Term Developmental Outcomes of Low Birth Weight Infants. Futur. Child..

[64] Van Buuren S. (2011). Mice: Multivariate Imputation by Chained Equations. J. Stat. Softw..

[65] Pruessner J.C., Kirschbaum C., Meinlschmid G., Hellhammer D.H. (2003). Two Formulas for Computation of the Area under the Curve Represent Measures of Total Hormone Concentration versus Time-Dependent Change. Psychoneuroendocrinology.

[66] Blaine B. (2018). Winsorizing. The SAGE Encyclopedia of Educational Research, Measurement, and Evaluation.

[67] Cohen J. (1988). Statistical Power Analysis for the Behavioral Sciences.

[68] Wright M.N., Ziegler A. (2015). Ranger: A Fast Implementation of Random Forests for High Dimensional Data in C++ and R. J. Stat. Softw..

[69] Mundfrom D., Perrett J., Schaffer J.R., Piccone A., Roozeboom M. (2006). Bonferroni Adjustments in Tests for Regression Coefficients. Mult. Linear Regres. Viewp..

[70] Vazquez E., Barranco A., Ramirez M., Gruart A., Delgado-Garcia J.M., Jimenez M.L., Buck R., Rueda R. (2016). Dietary 2′-Fucosyllactose Enhances Operant Conditioning and Long-Term Potentiation via Gut-Brain Communication through the Vagus Nerve in Rodents. PLoS ONE.

[71] Kuntz S., Kunz C., Borsch C., Vazquez E., Buck R., Reutzel M., Eckert G.P., Rudloff S. (2019). Metabolic Fate and Distribution of 2′-Fucosyllactose: Direct Influence on Gut Microbial Activity but Not on Brain. Mol. Nutr. Food Res..

[72] Sakanaka M., Hansen M.E., Gotoh A., Katoh T., Yoshida K., Odamaki T., Yachi H., Sugiyama Y., Kurihara S., Hirose J. (2019). Evolutionary Adaptation in Fucosyllactose Uptake Systems Supports Bifidobacteria-Infant Symbiosis. Sci. Adv..

[73] Ojima M.N., Asao Y., Nakajima A., Katoh T., Kitaoka M., Gotoh A., Hirose J., Urashima T., Fukiya S., Yokota A. (2022). Diversification of a Fucosyllactose Transporter within the Genus Bifidobacterium. Appl. Environ. Microbiol..

[74] Matsuki T., Yahagi K., Mori H., Matsumoto H., Hara T., Tajima S., Ogawa E., Kodama H., Yamamoto K., Yamada T. (2016). A Key Genetic Factor for Fucosyllactose Utilization Affects Infant Gut Microbiota Development. Nat. Commun..

[75] Planbureau S.e.C., Roeters A. (2019). Looking after the Household and Family Care. Time Use in The Netherlands.

[76] Liu F., Li P., Chen M., Luo Y., Prabhakar M., Zheng H., He Y., Qi Q., Long H., Zhang Y. (2017). Fructooligosaccharide (FOS) and Galactooligosaccharide (GOS) Increase Bifidobacterium but Reduce Butyrate Producing Bacteria with Adverse Glycemic Metabolism in Healthy Young Population. Sci. Rep..

[77] Borewicz K., Suarez-Diez M., Hechler C., Beijers R., de Weerth C., Arts I., Penders J., Thijs C., Nauta A., Lindner C. (2019). The Effect of Prebiotic Fortified Infant Formulas on Microbiota Composition and Dynamics in Early Life. Sci. Rep..

[78] Pisa E., Martire A., Chiodi V., Traversa A., Caputo V., Hauser J., Macrì S. (2021). Exposure to 3′sialyllactose-poor Milk during Lactation Impairs Cognitive Capabilities in Adulthood. Nutrients.

[79] Fleming S.A., Chichlowski M., Berg B.M., Donovan S.M., Dilger R.N. (2018). Dietary Sialyllactose Does Not Influence Measures of Recognition Memory or Diurnal Activity in the Young Pig. Nutrients.

[80] Mudd A.T., Fleming S.A., Labhart B., Chichlowski M., Berg B.M., Donovan S.M., Dilger R.N. (2017). Dietary Sialyllactose Influences Sialic Acid Concentrations in the Prefrontal Cortex and Magnetic Resonance Imaging Measures in Corpus Callosum of Young Pigs. Nutrients.

[81] Jacobi S.K., Yatsunenko T., Li D., Dasgupta S., Yu R.K., Berg B.M., Chichlowski M., Odle J. (2016). Dietary Isomers of Sialyllactose Increase Ganglioside Sialic Acid Concentrations in the Corpus Callosum and Cerebellum and Modulate the Colonic Microbiota of Formula-Fed Piglets. J. Nutr..

[82] Martorell R. (2017). Improved Nutrition in the First 1000 Days and Adult Human Capital and Health. Am. J. Hum. Biol..

[83] Hodel A.S., Senich K.L., Jokinen C., Sasson O., Morris A.R., Thomas K.M. (2017). Early Executive Function Differences in Infants Born Moderate-to-Late Preterm. Early Hum. Dev..

[84] Copeland A., Silver E., Korja R., Lehtola S.J., Merisaari H., Saukko E., Sinisalo S., Saunavaara J., Lähdesmäki T., Parkkola R. (2021). Infant and Child MRI: A Review of Scanning Procedures. Front. Neurosci..

[85] Rankin M.W., Jimenez E.Y., Caraco M., Collinson M., Lostetter L., DuPont T.L. (2016). Validation of Test Weighing Protocol to Estimate Enteral Feeding Volumes in Preterm Infants. J. Pediatr..

[86] Haase B., Barreira J., Murphy P.K., Mueller M., Rhodes J. (2009). The Development of an Accurate Test Weighing Technique for Preterm and High-Risk Hospitalized Infants. Breastfeed. Med..

