# Supplementary material for: Fucosylated Human Milk Oligosaccharides during the First 12 Postnatal Weeks Are Associated with Better Executive Functions in Toddlers
Source: Nutrients. 2023 Mar 17;15(6):1463. doi: 10.3390/nu15061463 (PMC10057664; doi:10.3390/nu15061463)
Supplement: Supplementary file 1 [file nutrients-15-01463-s001.zip › nutrients-2237227-supplementary.pdf]

nutrients MDPI

*Supplementary Materials*

# Fucosylated Human Milk Oligosaccharides during the First 12 Postnatal Weeks Are Associated with Better Executive Functions in Toddlers

**Table S1.** Associations between HMOs and HMO groups and measures of executive functioning including partially breastfed infants.

| | | Estimate (95% CI) | Standard error | *p*-value |
|---|---|---|---|---|
| BRIEF-P Model 1 | Intercept | 65.07 (40.01 - 90.12)*** | 12.52 | 0.00 |
| | 2′FL | -0.78 (-2.66 - 1.10) | 0.94 | 0.41 |
| | 6′SL | -22.34 (-63.77 - 19.08) | 20.69 | 0.28 |
| | 3′SL | 34.57 (-11.013 - 80.15) | 22.77 | 0.13 |
| | BRIEF-A | 0.20 (0.006 - 0.40)* | 0.10 | 0.04 |
| BRIEF-P Model 2 | Intercept | 139.06 (73.73 - 204.38)*** | 32.64 | 0.00 |
| | Fucosylated HMOs | -0.62 (-1.78 - 0.54) | 0.58 | 0.29 |
| | Sialylated HMOs | -14.24 (-28.45 - -0.02)* | 7.10 | 0.05 |
| | BRIEF-A | 0.19 (0.002 - 0.38)* | 0.09 | 0.05 |
| REEF Model 1 | Intercept | 166.97 (132.28 - 201.66) | 17.34 | 0.00 |
| | 2′FL | 3.87 (-0.19 - 7.93)[ł] | 2.03 | 0.06 |
| | 6′SL | -40.99 (-130.72 - 48.74) | 44.84 | 0.36 |
| | 3′SL | 1.18 (-97.01 - 99.36) | 49.07 | 0.98 |
| REEF Model 2 | Intercept | 88.35 (-39.60 - 216.30) | 63.97 | 0.17 |
| | Fucosylated HMOs | 2.19 (-0.44 - 4.82) | 1.31 | 0.10 |
| | Sialylated HMOs | 1.94 (-29.79 - 33.67) | 15.86 | 0.90 |
| Inhibitory control Model 1 | Intercept | 1.01 (0.22 - 1.80) | 0.40 | 0.01 |
| | 2′FL | 0.01 (-0.05 - 0.07) | 0.03 | 0.75 |
| | 6′SL | -0.94 (-2.25 - 0.37) | 0.65 | 0.16 |
| | 3′SL | 0.88 (-0.55 - 2.32) | 0.72 | 0.22 |
| | BRIEF-A | -0.01 (-0.01 - -0.002)* | 0.003 | 0.01 |
| Inhibitory control Model 2 | Intercept | 1.54 (-0.57 - 3.65) | 1.05 | 0.15 |
| | Fucosylated HMOs | 0.02 (-0.02 - 0.06) | 0.02 | 0.29 |
| | Sialylated HMOs | -0.27 (-0.73 - 0.19) | 0.23 | 0.24 |
| | BRIEF-A | -0.01 (-0.01 - -0.002)* | 0.003 | 0.01 |

Note that the analyses were performed on data including partially breastfed infants, *n* = 63. The REEF models did not include confounders as none of the potential confounders correlated with the REEF. The BRIEF-P is reverse coded to correspond with the other executive functions and inhibition measures (i.e. higher BRIEF-P scores indicate better executive functions). All HMOs and HMO groups mentioned in this table are the Area Under the Curve. [ł]: $p<0.1$, *: $p<0.05$, ***: $p<0.001$.

**Table S2.** Multiple logistic regression results between the HMOs and HMO groups and the BRIEF-P including partially breastfed infants.

| | | **Estimate (95% CI)** | **Standard error** | ***p*-value** |
|---|---|---|---|---|
| BRIEF-P Model 1 | Intercept | -2.79 (-7.51 – 1.51) | 2.25 | 0.23 |
| | 2′FL | - 0.03 (-0.63 -0.47) | 0.26 | 0.27 |
| | 6′SL | -10.36 (-26.00 - 1.86) | 6.80 | 0.65 |
| | 3′SL | -12.84 (-44.72 - 11.80) | 13.73 | 0.54 |
| | BRIEF-A | 0.03 (-0.02 - 0.10) | 0.03 | 0.14 |
| BRIEF-P Model 2 | Intercept | 3.31 (-10.17 – 17.88) | 6.97 | 0.64 |
| | Fucosylated HMOs | -0.04 (-0.42 - 0.27) | 0.16 | 0.27 |
| | Sialylated HMOs | -1.16 (-7.71 - 5.07) | 3.14 | 0.61 |
| | BRIEF-A | 0.03 (-0.01 - 0.09) | 0.02 | 0.17 |

Note that the analyses were performed on data including partially breastfed infants, $n$ = 63. All HMOs and HMO groups mentioned in this table are the Area Under the Curve. BRIEF-P coded as: 1, representing the high executive functions group and 0, representing the low executive functions group. Hence, positive values indicate a positive association between higher levels of HMOs and high executive functions.
